# Human Brucellosis in Maghreb: Existence of a Lineage Related to Socio-Historical Connections with Europe

**DOI:** 10.1371/journal.pone.0115319

**Published:** 2014-12-17

**Authors:** Nedjma Lounes, Moulay-Ali Cherfa, Gilles Le Carrou, Abdellah Bouyoucef, Maryne Jay, Bruno Garin-Bastuji, Virginie Mick

**Affiliations:** 1 EU/OIE/FAO & National Reference Laboratory for Brucellosis, Animal Health Laboratory, Paris-Est University/Anses, Maisons-Alfort, France; 2 Higher National Veterinary School (ENSV), Algiers, Algeria; 3 Institute of Veterinary Sciences, Saad Dahleb University, Blida, Algeria; Institut National de la Recherche Agronomique, France

## Abstract

Despite control/eradication programs, brucellosis, major worldwide zoonosis due to the *Brucella* genus, is endemic in Northern Africa and remains a major public health problem in the Maghreb region (Algeria/Morocco/Tunisia). *Brucella melitensis* biovar 3 is mostly involved in human infections and infects mainly small ruminants. Human and animal brucellosis occurrence in the Maghreb seems still underestimated and its epidemiological situation remains hazy. This study summarizes official data, regarding *Brucella melitensis* infections in Algeria, from 1989 to 2012, with the purpose to provide appropriate insights concerning the epidemiological situation of human and small ruminant brucellosis in Maghreb. Algeria and Europe are closely linked for historical and economical reasons. These historical connections raise the question of their possible impact on the genetic variability of *Brucella* strains circulating in the Maghreb. Other purpose of this study was to assess the genetic diversity among Maghreb *B. melitensis* biovar 3 strains, and to investigate their possible epidemiological relationship with European strains, especially with French strains. A total of 90 *B. melitensis* biovar 3 Maghreb strains isolated over a 25 year-period (1989–2014), mainly from humans, were analysed by MLVA-16. The obtained results were compared with genotypes of European *B. melitensis* biovar 3 strains. Molecular assays showed that Algerian strains were mainly distributed into two distinct clusters, one Algerian cluster related to European sub-cluster. These results led to suggest the existence of a lineage resulting from socio-historical connections between Algeria and Europe that might have evolved distinctly from the Maghreb autochthonous group. This study provides insights regarding the epidemiological situation of human brucellosis in the Maghreb and is the first molecular investigation regarding *B. melitensis* biovar 3 strains circulating in the Maghreb.

## Introduction

Brucellosis, due to the *Brucella* genus, is a major worldwide zoonosis. Despite eradication programs, the disease remains endemic in many regions of the world, with predominance in the Mediterranean Basin, especially in Maghreb (Algeria, Morocco and Tunisia), Middle East, Africa, western Asia, Central and South America. The global burden of human brucellosis remains important: the World Health Organization (WHO) estimates that the infection causes more than 500,000 infections per year worldwide [1], with an important travel-association [2]. The observed incidence in the endemic areas can range from <0.03 to>200 per 100,000 population. Nevertheless, if human brucellosis is a notifiable disease in most countries, official reports do not exactly reflect the number of persons affected every year (clinical polymorphism, political management, *etc*.) and WHO considers that the real incidence of this disease is 10–25 times superior to the notified [3].

Brucellosis affects wild and domestic mammals, especially cattle, small ruminants and swine, causing abortion and reduced fertility. The disease is transmitted to human through ingestion of contaminated dairy products (raw milk and unpasteurized cheeses) [2], as well as by direct contacts (cutaneous/mucous and aerosol inhalation) with infected animals or biological materials (carcass, abortion products, clinical samples).

Although animal and human brucellosis have been reported for a long time in Africa, the brucellosis occurrence in Maghreb was underestimated and its epidemiological situation remains poorly documented. In 1982, the Food and Agriculture Organization of the United Nations (FAO) stated the absence of *Brucella melitensis* in Algeria and in Morocco and its sporadic occurrence in Tunisia in goats and humans [4]. However, from the mi-1980s-1990s, the increase of human brucellosis in Algeria [5], Morocco [6] and Tunisia [7], [8] alerted the health authorities to the existence of an animal reservoir. *Brucella melitensis* infects mainly sheep and goats and is the major responsible for human infections, especially the predominant biovar (bv) 3 in Mediterranean countries [9]–[11].

Algeria and Morocco are important breeding countries, with a significant number of small ruminants (>22 million heads), while Tunisia has almost 9 million of sheep and goats [12]. Despite a lack of epidemiological data, it is admitted the disease is endemic in Maghreb, with brucellosis prevalence in small ruminants ranging from 0.1% for Morocco to 6% and 7.5%, respectively for Algeria and Tunisia [13].

The occurrence of human brucellosis depends largely on the animal reservoir. For preventing human brucellosis, the most efficient approach is the control and elimination of the animal infection. In Algeria, Morocco and Tunisia, similar control and eradication programs against brucellosis in small ruminants are applied, based on various strategies: mass vaccination in Algeria and Tunisia and authorized in Morocco and/or testing and slaughter of infected animals [9], [13]–[14]. However, in these areas where the animal infection is not yet controlled, the heat treatment (pasteurization) of dairy products is not systematic and certain food habits and faiths (consumption of raw milk/cheese) and/or inadequate hygienic practices related to poverty increase transmission to humans [15].

In non-endemic countries with controlled animal infection, human brucellosis occurrence is mainly due to imported cases from endemic areas, including Maghreb. France is in this case since it has been officially-free from bovine brucellosis since 2005 and no case has been reported in domestic and wild ruminants since 2003, except the recent outbreak in Haute-Savoie [16].

Molecular typing methods can be used for trace-back and trace-forward analysis which may help to identify infection origin and spreading route. The genetic diversity of *Brucella* strains isolated from human and animal infections has not yet been investigated in countries of Maghreb.

In the present study, the MLVA-16 (Multiple-Locus Variable-number tandem-repeat Analysis) assay, optimal strategy of *Brucella* molecular typing [17], was applied (*i*) to investigate epidemiological relationships among human and animal brucellosis isolates collected from different countries of Maghreb over 25 years (1989–2014), (*ii*) to determine the most common genotypes among *Brucella* strains in this region and (*iii*) to investigate their possible epidemiological relationship with European strains, especially with French strains.

## Materials and Methods

### Data source

Epidemiological data were extracted from human brucellosis cases occurred during 1990–2012 in France and during 1989–2011 in Algeria, reported respectively from the French institute for public health surveillance (InVS) and the Algerian institute for public health (INSP), where brucellosis is a notifiable disease, as well as from EFSA data [15], [18]–[19]. Population data were obtained from the French Institute of Statistics and Economic studies [20]. The number of imported cases to France from Maghreb was completed with data from InVS. Regarding brucellosis in small ruminants, data were extracted from OIE interfaces: Handistatus II system [21] for the years before 2005 screening the caprine and ovine brucellosis excluding *B. ovis* [OIE code B152], and WAHIS (World Animal Health Information System) [22] providing data from 2006, selecting “Brucellosis (*Brucella melitensis*) (2006-)”. OIE defines the “cases” as the animals affected by the disease, *i.e.* sick animals and the animals that died from the disease.

### *Brucella* strains

Ninety strains, isolated mainly from humans and also from animals (one bovine and two ovine strains), from 1989 to 2014 (S1 Table), were obtained from the ANSES collection, except for one bovine strain, obtained from a routine epidemiological veterinary investigation conducted in Algeria in 2011–2014 (Lounes N, personal data). All isolates were confirmed as *B. melitensis* bv 3 using the classical microbiological methods, based on CO_2_ requirement, H_2_S production, urea hydrolysis, agglutination with monospecific sera, fuchsin and thionin dye sensitivity and phage typing [23].

Although most strains were isolated in France, all have a Maghreb geographical origin: 56 strains isolated from Algeria, including some strains isolated from a same patient (2 patients with two strains each: M021/M022 and M069/M070), as well as from a mother and her son (M029/M030) or from the same family (M060/M062), with 3 laboratory-acquired infections (M012/M020, M054/M055/M059, M071/M072), 14 strains isolated from Morocco (including from one husband and his wife: M035/M036) with 1 strain from laboratory-acquired infection (M001/M007), 13 strains isolated from Tunisia (including two strains from a same patient: M044/M049), and 7 strains from *largo sensu* Maghreb (when the country origin was not clearly determined) (S1 Table) with 1 related strain from laboratory-acquired infection (M024/M025).

Two reference strains *B. melitensis* bv 1 str. 16M (M093) and *B. melitensis* bv 3 str. Ether (M092), as well as one *B. melitensis* bv 3 human isolate from Turkey (M091) were included as controls for molecular analysis.

### Molecular approaches

*Brucella* DNA sample was prepared using the High Pure PCR Template Preparation Kit (Roche Diagnostics, France), according to the manufacturer's instructions. A *Brucella* genus-specific real-time PCR (RT-PCR) (Applied Biosystems, France) was performed on DNA extracts, as previously described [24]. The *B. melitensis* bv 3 strains were characterized by MLVA-16, as previously described [17], [25].

### Data analysis

Fragment sizes converted to repeat unit (U) numbers [25] were analyzed as a character data set, using BioNumerics v6.6 (Applied Maths, Belgium). According to the molecular evolution, weights were assigned to the distinct panels (weights of 2, 1 and 0.1, respectively for the panel 1, panel 2A and panel 2B) [17]. MLVA genotypes were identified into the database *Brucella* 2012 (Orsay, France) (http://mlva.u-psud.fr/mlvav4/genotyping/). The cluster analysis was performed using the UPGMA (Unweighted Pair Group Method Algorithm) algorithm or using a Minimum Spanning Tree (MST) with Euclidean distance matrices. Regarding the wide world circulation of *B. melitensis* bv 3, especially in Europe and in Maghreb, the cluster analysis was extended to 507 published MLVA patterns, including MLVA genotypes of the Maghreb strains investigated in this study [17], [25]–[31].

The Hunter & Gaston diversity index (HGDI) of each locus was determined (http://www.hpa-bioinformatics.org.uk/cgi-bin/DICI/DICI.pl). The HGDI values can range from 0.0 (no diversity) to 1.0 (complete diversity).

## Results and Discussion

This study is the first molecular investigation regarding the *Brucella* strains circulating in Maghreb (Algeria, Morocco and Tunisia) over a 25 year-period (1989–2014), including strains isolated from patients diagnosed in France, but whose contamination origin was undoubtedly Maghreb.

### Overview of human and animal brucellosis in Maghreb: focus on Algeria

Brucellosis is endemic in the Mediterranean basin, especially in the Northern African countries [1], [32]. Until the 1980s, the epidemiological situation was not well known in Maghreb and cases of human brucellosis were rarely reported in Algeria, Morocco and Tunisia because the infection was misdiagnosed or ignored, despite an important animal reservoir [12]. Since the middle of 1980s, several outbreaks, as the Ghardaïa (Southern Algeria) outbreak in 1984 resulting in more than 600 human cases [33], the Gafsa (Southern Tunisia) outbreak in 1991 with more than 400 human cases [34], have shaken the public health services and led to the implementation of control and eradication measures, regarding animal brucellosis as well as human brucellosis [14]. Despite these adopted control measures, the disease is still present [35], and although brucellosis is a notifiable disease in Maghreb countries, incidence is still underestimated and/or under-reported, as the low human incidence suggests (8.43 annual cases per 100,000 population in Algeria in 2006) [1].

**Figure 1:**
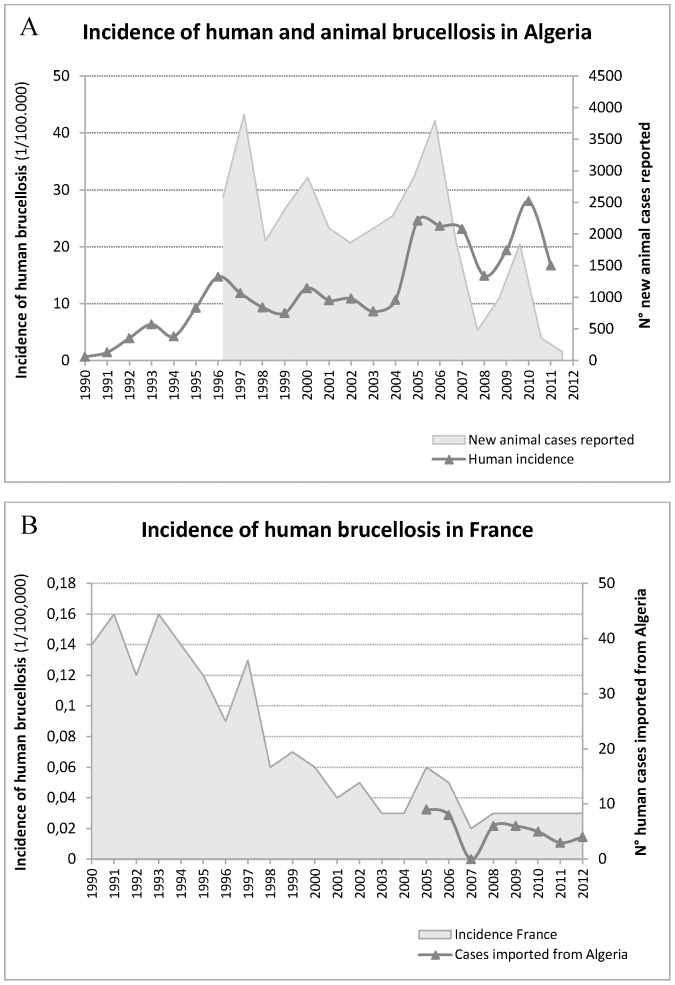
Incidence of *B. melitensis* human and animal brucellosis in Algeria, *vs* incidence of human brucellosis in France and number of human cases imported from Algeria to France. A: Incidence of human and animal brucellosis in Algeria (1/100,000 inhabitants); B: Incidence of human brucellosis in France (1/100,000 inhabitants) and number of human cases imported from Algeria to France The x-axis indicates the year. The y-axis represents (A) incidence of human brucellosis or number of new animal cases reported in Algeria and (B) incidence of human brucellosis in France or number of new human *B. melitensis* cases imported from Algeria to France.

Fig. 1A shows the *B. melitensis* incidence reported in Algeria both in humans and animals (S2 Table). The incidence of human brucellosis in Algeria shows an upward trend since 1990, with values ranging from 0.36 in 1989 to 16.76 per 100,000 inhabitants in 2011, reaching 23–24.6 in 2005–2007 and up to 28 in 2010. This increase could be the result of diagnosis and surveillance improvement. As expected, the numbers of new animal and human cases reported from Algeria follow a similar trend, highlighting the impact of the animal reservoir on the Public Health (Fig. 1A). Human incidence might reflect the true epidemiological situation of brucellosis in animals. However, while new annual human cases reported from Algeria are important, prevalence reported in small ruminants in Algeria remains low (in 2009 only 6% in sheep and goat [13]), questioning the reliability of the compulsory notification.

In France, where ruminant brucellosis has been eradicated since 2000s, incidence of human brucellosis followed a constant decline from 1990 to 2000 (Fig. 1B). The incidence rate is currently stable and lower than 0.04 per 100,000 inhabitants in 2012, with 23 reported cases (including 19 imported cases). Indeed, annual reported cases are mainly due to imported cases from endemic areas and are epidemiologically investigated by InVS to determine risk factors and the putative infection source (travel in endemic area, contaminated raw milk/cheese consumption, infected animal contacts…). These human cases imported from Maghreb, but diagnosed in France (Fig. 1B), are thus reported with certainty and their incidence curve shows the same pattern than human brucellosis in Algeria (Fig. 1). For social and historical reasons, migration flows from Algeria are important to France. Analysis of the epidemiological situation of brucellosis diagnosed in France, but originated from Maghreb, especially from Algeria, might reflect the epidemic features of human and animal brucellosis in Maghreb. Nevertheless, a time-lag of one year was observed between both curves (cases imported to France and Algerian incidence), suggesting an improved diagnosis in France.

In addition, WHO considers human brucellosis as a neglected disease of poverty [36]. The percentage of the population living in poverty is important in Maghreb (24% for Algeria population; 36 millions) [37]. Despite education campaigns, social and food habits remain, especially in rural areas, where the population lives in close contact with animals; consumption of unpasteurized products is responsible of 85% of human infections in Algeria [38]–[39].

### Genetic diversity of MLVA-16 molecular markers for the Maghreb strains

The HGDI for each MLVA-16 locus was calculated from 90 Maghreb *B. melitensis* bv 3 strains, and compared to published global HGDI data for *B. melitensis* bv 3 strains [31] (Table 1). HGDI values ranged from 0.000 to 0.022 in panel 1, from 0.022 to 0.585 in panel 2A and from 0.000 to 0.802 in panel 2B. The loci of panel 1, as well as the Bruce21 of panel 2A, with values closed to zero, present no discriminatory power for the epidemiological situation of the investigated strains. Interestingly, and contrary to published HGDI [40], HGDI values of Bruce30 (panel 2B) displayed no informational nature in this study, with HGDI values of 0.000.

**Table 1:** *B. melitensis* bv 3 HGDI values for each MLVA-16 genetic marker (from this study and from published data).

	Locus	HGDI from obtained results in this study				Global HGDI*			
		Diversity Index (*n = 90*)	Confidence Interval	K	max(pi)	Diversity Index (*n*)	Confidence Interval	K	max(pi)
**Panel 2B**	Bruce04	0.782	0.743–0.821	8	0.311	0.765 (*507*)	0.745–0.785	10	0.377
	Bruce07	0.800	0.767–0.834	7	0.311	0.676 (*507*)	0.641–0.710	10	0.518
	Bruce09	**0.802**	0.731–0.874	14	0.411	0.637 (*507*)	0.592–0.683	15	0.588
	Bruce16	**0.802**	0.770–0.834	7	0.300	**0.838** (*507*)	0.828–0.849	11	0.245
	Bruce30	0.000	0.000–0.077	1	1.000	0.693 (*507*)	0.664–0.721	7	0.480
**Panel 2A**	Bruce18	0.585	0.528–0.641	4	0.522	0.582 (*432*)	0.541–0.624	7	0.595
	Bruce19	0.542	0.475–0.609	3	0.589	0.577 (*432*)	0.540–0.615	7	0.583
	Bruce21	0.022	0.000–0.065	2	0.989	0.009 (*432*)	0.000–0.022	2	0.995
**Panel 1**	Bruce06	0.000	0.000–0.077	1	1.000	0.466 (*432*)	0.442–0.490	2	0.632
	Bruce08	0.022	0.000–0.065	2	0.989	0.085 (*432*)	0.049–0.121	4	0.956
	Bruce11	0.000	0.000–0.077	1	1.000	0.000 (*432*)	0.000–0.017	1	1.000
	Bruce12	0.000	0.000–0.077	1	1.000	0.093 (*432*)	0.056–0.131	3	0.951
	Bruce42	0.022	0.000–0.065	2	0.989	0.663 (*432*)	0.654–0.672	4	0.370
	Bruce43	0.000	0.000–0.077	1	1.000	0.504 (*432*)	0.479–0.529	4	0.595
	Bruce45	0.000	0.000–0.077	1	1.000	0.000 (*432*)	0.000–0.017	1	1.000
	Bruce55	0.000	0.000–0.077	1	1.000	0.466 (*432*)	0.442–0.490	2	0.632

Hunter Gaston Diversity Index (HGDI): Measure of the variation of the number of repeats at each locus. Ranges from 0.0 (no diversity) to 1.0 (complete diversity); Confidence Interval: Precision of the Diversity Index, expressed as 95% upper and lower boundaries; K: Number of different repeats present at this locus in this sample set; max(pi): Fraction of samples that have the most frequent repeat number in this locus (range 0.0 to 1.0); n: strains number according to the considered locus; in bold: locus more variable. *: [17], [25]–[31].

As expected with the global data, the most variable loci (with HGDI values given in brackets) belonged to panel 2B: Bruce09 (0.802), Bruce16 (0.802), Bruce07 (0.800) and Bruce04 (0.782).

### Genotypes and clusters

All investigated strains (n = 90), excluding the control strains (*B. melitensis* bv 1 16 M and *B. melitensis* bv 3 Ether, as well as the Turkey strain M091), belonged to the West Mediterranean group (S1 Table) [17]. All Maghreb strains harbored a MLVA-8 (only panel 1) genotype 51, except one (M002) with the genotype 88 and one (M035) showing a new genotype (S1 Table). They fell into 10 MLVA-11 (panel 1 and panel 2A) genotypes: predominant genotypes 92 and 94, each with 29 strains (32.2%), followed by the genotype 96 (16 strains; 17.8%), other previously described: 91 (4 strains; 4.5%), 155 (1 strain; 1.1%), a genotype matching with the Italian strain #11789 [41] (2 strain; 2.2%) and four new genotypes (5 strains, 5.6%; 2 strains, 2.2%; 1 strain, 1.1%; 1 strain, 1.1%) (S1 Table). Interestingly, the genotype 94 seems to be shared only by the Maghreb strains, although in the public database *Brucella* 2012, some strains identified as non Maghreb strains harbor this genotype 94, e.g. BCCN#03-13, a strain isolated in 2003 in France [17]. These strains might be epidemiologically misidentified.

**Figure 2:**
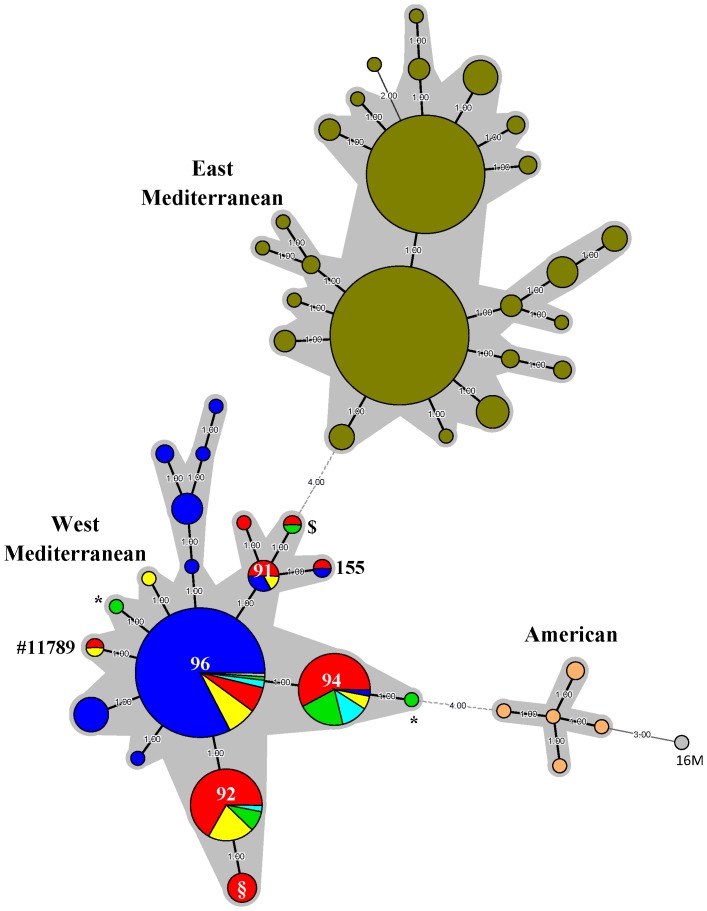
MST of clustered MLVA-11 genotypes of the worldwide *B. melitensis* bv 3 isolates. The MST was constructed with a categorical coefficient (with panel weights). Size of circles reflects the number of isolates with a particular MLVA genotype. Width of the line reflects the genetic distance between the genotypes (heavy short lines connect SLVs, thin longer lines connect DLVs). Each country/group is assigned a different colour, *i.e.* isolates from Algeria are coloured in red, isolates from Morocco in green, isolates from Tunisia in yellow, isolates from *largo sensu* Maghreb in light blue, West Mediterranean isolates from Europe in blue, East Mediterranean isolates (n = 267) in khaki, American isolates in orange and the *B. melitensis* bv 1 reference strain 16 M in grey. Regarding the West Mediterranean group, the MLVA-11 genotypes identified in this study were indicated (S1 Table).

As previously described, MLVA-11 allows assessing global phylogeographic relationship [30]. MLVA-11 cluster analysis based on 497 worldwide *B. melitensis* bv 3 strains, including the Maghreb strains investigated in this study, was performed (Fig. 2). The strains were divided into three clusters: West Mediterranean, East Mediterranean and American clusters [17]. As previously described, the East Mediterranean group essentially contained the strains isolated from Turkey (including M091) [42], China [40], [43], Germany and Greece, while the West Mediterranean group was constituted by the European strains (Italy, Spain, France, Switzerland) [26]–[27], [30]–[31] and the Maghreb strains (Algeria, Morocco, Tunisia) (this study).

**Figure 3:**
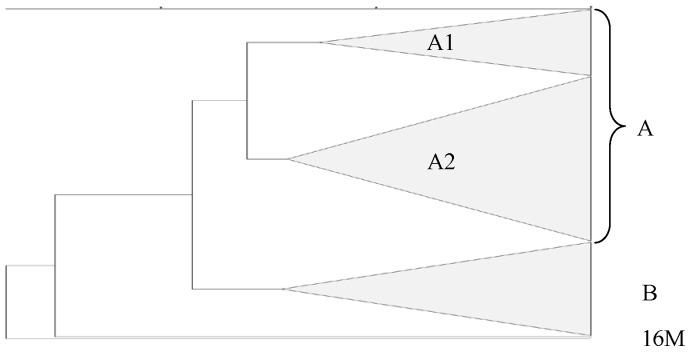
Concatenated dendrogram of clustered MLVA-16 genotypes of Europe and Maghreb isolates. The dendrogram was constructed with a categorical coefficient and UPGMA algorithm. Clusters are coded as A and B, and sub-clusters as A1–A2. MLVA-16 genotypes of all investigated strains and strain details are shown in S1 Figure.

Phylogenetic relationships of the Maghreb *B. melitensis* bv 3 isolates were revealed by a concatenated dendrogram based on MLVA-16 genotyping (Fig. 3). MLVA-16 genotypes of the European strains (France, Spain, Italy) were added in the distance matrix construction ([26]–[27], [30]–[31] and personal results). The detailed dendrogram is shown in the S1 Figure. The investigated strains in this study were distributed into 55 MLVA-16 genotypes. Excluding the *B. melitensis* bv 1 16 M reference strain (cluster D) and the Algerian human strain M038 (cluster C), all Maghreb and Europe *B. melitensis* bv 3 strains (n = 215) clustered into 2 clusters, designated A and B, with respectively 153 strains (71.2%) and 62 strains (28.8%) (S1 Figure).

Interestingly, with a >98% similarity cut-off, the Algeria strains were distributed into two distinct clusters (clusters A and B), one Algerian cluster (subcluster A1 with 44 strains mainly originated from Maghreb) related to the Moroccan strains and very close to the European cluster (subcluster A2 with 109 strains mostly isolated from Europe), while the other Algerian cluster (cluster B) was closer to the Tunisian strains (Fig. 3). These results suggest that these two sublineages could reflect the existence of an autochthonous lineage from Maghreb (cluster B), as well as an “historical lineage” (cluster A), resulting from historical and economical connections between Algeria and Europe. Previous Pulsed-Field Gel Electrophoresis studies performed from France and Algeria strains, after *Xho*I and *Xba*I restrictions, already highlighted the existence of two patterns: an autochthonous Algerian pattern and a “colonial” pattern [44]. Former movements of infected animals from Europe to Maghreb (*e.g.* brucellosis seropositive pregnant cows from France in the 1970's, goats from Malta or Spain at the end 19^th^ century…) [45] are described and might be involved in the existence of this imported pattern. Our current investigation, based on more discriminative methods, seems to support this assumption.

Furthermore, these results highlighted some geographical patterns, probably related to socioeconomic links between Tunisia/Algeria and Morocco/Algeria (*e.g*. bilateral trade; breeding systems: grazing, transhumance; uncontrolled animal movement across borders…). However, so far, the lack of specific geographical location of studied strains, as well as insufficient sampling from Morocco and Tunisia do not allow the full confirmation of this scenario.

Some epidemiologically related strains, *i.e*. isolated from a same patient, from some members of the same family and from patient/laboratory-acquired infection, were investigated. As expected, the most related strains (M001/M007; M021/M022; M024/M025; M029/M030; M044/M049; M054/M055/M059; M060/M062; M069/M070; M071/M072) harbored a strictly identical MLVA-16 genotype. However, it was more surprising to note that some epidemiologically related strains showed small variations in their MLVA-16 patterns. The most important difference concerns the genotypes of strains isolated from a husband (M035) and his wife (M036), with some differences on each panel: 1 U on Bruce42 (panel 1), 2 U on Bruce18 (panel 2A) and 4 U on Bruce09 (panel 2B), suggesting distinct infection sources. A similar result was observed for strains isolated from a patient and a technician contaminated directly from a sample of this patient (M012/M020) harbored distinct genotypes, with small variations in the number of repeats (1 U) on two loci (Bruce04 and Bruce07) of the hypervariable panel 2B.

## Conclusions

Although Maghreb is an endemic area with an important animal reservoir, the epidemiological situation remains hazy. Incidence of human and animal brucellosis is yet underestimated and under reported in these countries.

This study provides insights regarding the epidemiological situation of human brucellosis in Maghreb and describes for the first time the molecular characterization of *B. melitensis* strains circulating in Algeria, Morocco and Tunisia. The current molecular typing method with high resolution discrimination MLVA-16 allows clustering the Maghreb strains into two geographical lineages, suggesting the existence of a Maghreb autochthonous group and a lineage resulting from socio-historical connections with Europe.

## Supporting Information

S1 Figure **Detailed dendrogram of clustered MLVA-16 genotypes of the Maghreb and Europe** ***B. melitensis*** **bv 3 isolates.** The dendrogram was constructed with a categorical coefficient and UPGMA algorithm. Clusters are coded as A–D, and sub-clusters as A1–A2. Each country is assigned a different colour, *i.e.* isolates from Algeria are coloured in red and isolates from Morocco in green. MLVA-16 data for each strain were shown.
(DOCX)

S1 Table ***B. melitensis*** **biovar 3 strains isolated from different geographical origins, investigated in this study and MLVA genotype results.**
(DOCX)

S2 Table **Data of small ruminant brucellosis reported in Algeria.**
(DOCX)
