# Supplementary material for: Human Brucellosis in Maghreb: Existence of a Lineage Related to Socio-Historical Connections with Europe
Source: PLoS One. 2014 Dec 17;9(12):e115319. doi: 10.1371/journal.pone.0115319 (PMC4269447; doi:10.1371/journal.pone.0115319)
Supplement: S1 Figure — Detailed dendrogram of clustered MLVA-16 genotypes of the Maghreb and Europe B. melitensis bv 3 isolates. The dendrogram was constructed with a categorical coefficient and UPGMA algorithm. Clusters are coded as A–D, and sub-clusters as A1–A2. Each country is assigned a different colour, i.e. isolates from Algeria are coloured in red and isolates from Morocco in green. MLVA-16 data for each strain were shown. (DOCX) [file pone.0115319.s001.docx]

**Fig. S1**: Dendrogram of clustered MLVA-16 genotypes of Europe and Maghreb isolates

(Euclidian distance, UPGMA algorithm)
