# Supplementary material for: Human Brucellosis in Maghreb: Existence of a Lineage Related to Socio-Historical Connections with Europe
Source: PLoS One. 2014 Dec 17;9(12):e115319. doi: 10.1371/journal.pone.0115319 (PMC4269447; doi:10.1371/journal.pone.0115319)
Supplement: S1 Table — B. melitensis biovar 3 strains isolated from different geographical origins, investigated in this study and MLVA genotype results. (DOCX) [file pone.0115319.s002.docx]

Supplementary Table S1: ***Brucella melitensis* biovar 3 strains isolated from different geographical origins, investigated in this study and MLVA genotype results.**

| **Key** | **Id Strain** | **Links** | **Year** | **Host** | **Country** | **Genus** | **Species** | **Bv** | **Group** | **MLVA8** | **MLVA11** | **MLVA-16** |
| --- | --- | --- | --- | --- | --- | --- | --- | --- | --- | --- | --- | --- |
| **M001** | 14-195-380 | M007 (patient) | 2014 | Human | Morocco (Technician) | *Brucella* | *melitensis* | 3 | West mediterranean | 51 | 94 | a |
| **M002** | 13-06-37 |  | 2013 | Human | Algeria | *Brucella* | *melitensis* | 3 | West mediterranean | 88 | 155 | * |
| **M003** | 13-55-202 |  | 2013 | Human | Algeria | *Brucella* | *melitensis* | 3 | West mediterranean | 51 | 91 | * |
| **M004** | 13-717-1579 |  | 2013 | Human | Algeria | *Brucella* | *melitensis* | 3 | West mediterranean | 51 | 94 | * |
| **M005** | 13-762-1656 |  | 2013 | Human | Morocco | *Brucella* | *melitensis* | 3 | West mediterranean | 51 | 94 | * |
| **M006** | 13-2348-3996 |  | 2013 | Human | Algeria | *Brucella* | *melitensis* | 3 | West mediterranean | 51 | 96 | * |
| **M007** | 13-3383-8396 | M001 (technician) | 2013 | Human | Morocco | *Brucella* | *melitensis* | 3 | West mediterranean | 51 | 94 | a |
| **M008** | 13-3841-9758 |  | 2013 | Human | Algeria | *Brucella* | *melitensis* | 3 | West mediterranean | 51 | 96 | * |
| **M009** | 12-0017-26 |  | 2012 | Human | Algeria | *Brucella* | *melitensis* | 3 | West mediterranean | 51 | 94 | * |
| **M010** | 12-0039-53 |  | 2012 | Human | Algeria | *Brucella* | *melitensis* | 3 | West mediterranean | 51 | 92 | * |
| **M011** | 12-2492-5347 |  | 2012 | Human | Algeria | *Brucella* | *melitensis* | 3 | West mediterranean | 51 | § | * |
| **M012** | 12-2906-6073 | M020 (patient) | 2012 | Human | Algeria (Technician) | *Brucella* | *melitensis* | 3 | West mediterranean | 51 | 94 | * |
| **M013** | 12-2916-6076 |  | 2012 | Human | Algeria | *Brucella* | *melitensis* | 3 | West mediterranean | 51 | 96 | * |
| **M014** | 12-3104-6836 |  | 2012 | Human | Tunisia | *Brucella* | *melitensis* | 3 | West mediterranean | 51 | 92 | * |
| **M015** | 12-3218-7014 |  | 2012 | Human | Morocco | *Brucella* | *melitensis* | 3 | West mediterranean | 51 | $ | * |
| **M016** | 12-4523-9117 |  | 2012 | Human | Algeria | *Brucella* | *melitensis* | 3 | West mediterranean | 51 | 94 | a |
| **M017** | 11-512-1322 |  | 2011 | Human | Tunisia | *Brucella* | *melitensis* | 3 | West mediterranean | 51 | #11789 ^&^ | * |
| **M018** | 11-1392-2557 |  | 2011 | Human | Tunisia | *Brucella* | *melitensis* | 3 | West mediterranean | 51 | 96 | 2006LeFlèche#086 ^&^ |
| **M019** | 11-1544-3038 |  | 2011 | Human | Algeria | *Brucella* | *melitensis* | 3 | West mediterranean | 51 | 92 | * |
| **M020** | 11-2478-4373 | M012 (technician) | 2011 | Human | Algeria | *Brucella* | *melitensis* | 3 | West mediterranean | 51 | 94 | * |
| **M021** | 11-2673-4772 | M022 (same patient) | 2011 | Human | Algeria | *Brucella* | *melitensis* | 3 | West mediterranean | 51 | § | b |
| **M022** | 11-2676-4776 | M021 (same patient) | 2011 | Human | Algeria | *Brucella* | *melitensis* | 3 | West mediterranean | 51 | § | b |
| **M023** | 27/GM |  | 2011 | Bovine | Algeria | *Brucella* | *melitensis* | 3 | West mediterranean | 51 | 94 | * |
| **M024** | 10-366-1509 | M025 (patient) | 2010 | Human | Maghreb (Technician) | *Brucella* | *melitensis* | 3 | West mediterranean | 51 | 94 | c |
| **M025** | 10-367-1510 | M024 (technician) | 2010 | Human | Maghreb | *Brucella* | *melitensis* | 3 | West mediterranean | 51 | 94 | c |
| **M026** | 10-394 |  | 2010 | Human | Algeria | *Brucella* | *melitensis* | 3 | West mediterranean | 51 | 91 | * |
| **M027** | 10-915 |  | 2010 | Human | Morocco | *Brucella* | *melitensis* | 3 | West mediterranean | 51 | 94 | * |
| **M028** | 10-1349 |  | 2010 | Human | Algeria | *Brucella* | *melitensis* | 3 | West mediterranean | 51 | 92 | * |
| **M029** | 10-1365 | M030 (son) | 2010 | Human | Algeria | *Brucella* | *melitensis* | 3 | West mediterranean | 51 | 94 | d |
| **M030** | 10-1366 | M029 (mother) | 2010 | Human | Algeria | *Brucella* | *melitensis* | 3 | West mediterranean | 51 | 94 | d |
| **M031** | 10-2445 |  | 2010 | Human | Algeria | *Brucella* | *melitensis* | 3 | West mediterranean | 51 | $ | * |
| **M032** | 09-497-1031 |  | 2009 | Human | Morocco | *Brucella* | *melitensis* | 3 | West mediterranean | 51 | 94 | * |
| **M033** | 09-521-1131 |  | 2009 | Human | Maghreb? (Technician) | *Brucella* | *melitensis* | 3 | West mediterranean | 51 | 96 | * |
| **M034** | 09-537-1148 |  | 2009 | Human | Morocco | *Brucella* | *melitensis* | 3 | West mediterranean | 51 | 92 | * |
| **M035** | 09-577-1245 | M036 (wife) | 2009 | Human | Morocco | *Brucella* | *melitensis* | 3 | West mediterranean | * | * | * |
| **M036** | 09-669-1401 | M035 (husband) | 2009 | Human | Morocco | *Brucella* | *melitensis* | 3 | West mediterranean | 51 | 92 | * |
| **M037** | 09-975 |  | 2009 | Human | Maghreb | *Brucella* | *melitensis* | 3 | West mediterranean | 51 | 92 | * |
| **M038** | 09-1057 |  | 2009 | Human | Algeria | *Brucella* | *melitensis* | 3 | West mediterranean | 51 | 96 | * |
| **M039** | 09-1079-2065 |  | 2009 | Human | Algeria | *Brucella* | *melitensis* | 3 | West mediterranean | 51 | 94 | * |
| **M040** | 09-1162-2209 |  | 2009 | Human | Algeria | *Brucella* | *melitensis* | 3 | West mediterranean | 51 | #11789 | * |
| **M041** | 09-1166-2214 |  | 2009 | Human | Morocco | *Brucella* | *melitensis* | 3 | West mediterranean | 51 | 94 | * |
| **M042** | 09-1517-2957 |  | 2009 | Human | Morocco? (Maghreb) | *Brucella* | *melitensis* | 3 | West mediterranean | 51 | * | * |
| **M043** | 09-1658-3209 |  | 2009 | Human | Algeria? (Maghreb) | *Brucella* | *melitensis* | 3 | West mediterranean | 51 | § | * |
| **M044** | 08-17-36 | M049 (same patient) | 2007 | Human | Tunisia | *Brucella* | *melitensis* | 3 | West mediterranean | 51 | 96 | e |
| **M045** | 08-98-205 |  | 2008 | Human | Tunisia | *Brucella* | *melitensis* | 3 | West mediterranean | 51 | 96 | * |
| **M046** | 08-983-1783 |  | 2008 | Human | Algeria | *Brucella* | *melitensis* | 3 | West mediterranean | 51 | 96 | * |
| **M047** | 08-2554-5444 |  | 2008 | Human | Algeria | *Brucella* | *melitensis* | 3 | West mediterranean | 51 | § | * |
| **M048** | 07-717-1483 |  | 2007 | Human | Morocco | *Brucella* | *melitensis* | 3 | West mediterranean | 51 | 96 | * |
| **M049** | 07-1183-2851 | M044 (same patient) | 2007 | Human | Tunisia | *Brucella* | *melitensis* | 3 | West mediterranean | 51 | 96 | e |
| **M050** | 07-1197-2873 |  | 2007 | Human | Tunisia | *Brucella* | *melitensis* | 3 | West mediterranean | 51 | 91 | * |
| **M051** | 07-1390 |  | 2007 | Human | Tunisia | *Brucella* | *melitensis* | 3 | West mediterranean | 51 | 92 | * |
| **M052** | 06-0157 |  | 2006 | Human | Tunisia | *Brucella* | *melitensis* | 3 | West mediterranean | 51 | 96 | * |
| **M053** | 06-1012-1697 |  | 2006 | Human | Algeria | *Brucella* | *melitensis* | 3 | West mediterranean | 51 | 92 | f |
| **M054** | 06-1352-2309 | M055 (Technician 1) ; M059 (Technician 2) | 2006 | Human | Algeria | *Brucella* | *melitensis* | 3 | West mediterranean | 51 | 92 | g |
| **M055** | 06-1353-2310 | M054 (patient);  M059 (Technician 2) | 2006 | Human | Algeria (Technician 1) | *Brucella* | *melitensis* | 3 | West mediterranean | 51 | 92 | g |
| **M056** | 06-1530 |  | 2006 | Human | Algeria | *Brucella* | *melitensis* | 3 | West mediterranean | 51 | 92 | g |
| **M057** | 06-1628-2750 |  | 2006 | Human | Tunisia | *Brucella* | *melitensis* | 3 | West mediterranean | 51 | 92 | f |
| **M058** | 06-1639 |  | 2006 | Human | Algeria | *Brucella* | *melitensis* | 3 | West mediterranean | 51 | 94 | f |
| **M059** | 06-1742 | M054 (patient);  M055 (Technician 1) | 2006 | Human | Algeria (Technician 2) | *Brucella* | *melitensis* | 3 | West mediterranean | 51 | 92 | g |
| **M060** | 06-2224 | M062 (same family) | 2006 | Human | Algeria | *Brucella* | *melitensis* | 3 | West mediterranean | 51 | 92 | h |
| **M061** | 06-2272-ganglions |  | 2006 | Human | Algeria | *Brucella* | *melitensis* | 3 | West mediterranean | 51 | 94 | * |
| **M062** | 06-2321 | M060 (same family) | 2006 | Human | Algeria | *Brucella* | *melitensis* | 3 | West mediterranean | 51 | 92 | h |
| **M063** | 06-2660 |  | 2006 | Human | Algeria | *Brucella* | *melitensis* | 3 | West mediterranean | 51 | 92 | * |
| **M064** | 05-2077 |  | 2005 | Human | Tunisia | *Brucella* | *melitensis* | 3 | West mediterranean | 51 | 92 | * |
| **M065** | 05-2156 |  | 2005 | Human | Algeria | *Brucella* | *melitensis* | 3 | West mediterranean | 51 | 92 | * |
| **M066** | 05-2225 |  | 2005 | Human | Tunisia | *Brucella* | *melitensis* | 3 | West mediterranean | 51 | 94 | * |
| **M067** | 05-2698 |  | 2005 | Human | Algeria | *Brucella* | *melitensis* | 3 | West mediterranean | 51 | 96 | * |
| **M068** | 05-2833 |  | 2005 | Human | Algeria | *Brucella* | *melitensis* | 3 | West mediterranean | 51 | 92 | * |
| **M069** | 05-2951 | M070 (same patient) | 2005 | Human | Algeria | *Brucella* | *melitensis* | 3 | West mediterranean | 51 | 92 | 2007AlDahouk#018^l &^ |
| **M070** | 05-3028 | M069 (same patient) | 2005 | Human | Algeria | *Brucella* | *melitensis* | 3 | West mediterranean | 51 | 92 | 2007AlDahouk#018^l &^ |
| **M071** | 05-3156 | M072 (Technician) | 2005 | Human | Algeria | *Brucella* | *melitensis* | 3 | West mediterranean | 51 | 94 | i |
| **M072** | 05-3434 | M071 (patient) | 2005 | Human | Algeria (Technician) | *Brucella* | *melitensis* | 3 | West mediterranean | 51 | 94 | i |
| **M073** | 05-3619 |  | 2005 | Human | Algeria | *Brucella* | *melitensis* | 3 | West mediterranean | 51 | 92 | 2007AlDahouk#025 ^&^ |
| **M074** | 05-4323 |  | 2002 | Human | Maghreb? (Technician) | *Brucella* | *melitensis* | 3 | West mediterranean | 51 | 96 | * |
| **M075** | 04-1953 |  | 2004 | Human | Tunisia | *Brucella* | *melitensis* | 3 | West mediterranean | 51 | 96 | * |
| **M076** | 04-3016 |  | 2004 | Human | Algeria | *Brucella* | *melitensis* | 3 | West mediterranean | 51 | 92 | j |
| **M077** | 04-3189 |  | 2004 | Human | Algeria? (Maghreb) | *Brucella* | *melitensis* | 3 | West mediterranean | 51 | 94 | 2006LeFlèche#087 ^&^ |
| **M078** | 04-3519 |  | 2004 | Human | Algeria | *Brucella* | *melitensis* | 3 | West mediterranean | 51 | 91 | * |
| **M079** | 04-3960-6000 |  | 2004 | Human | Algeria | *Brucella* | *melitensis* | 3 | West mediterranean | 51 | 92 | j |
| **M080** | 03-524 |  | 2002 | Human | Algeria | *Brucella* | *melitensis* | 3 | West mediterranean | 51 | 92 | * |
| **M081** | 03-2824 |  | 2003 | Human | Algeria | *Brucella* | *melitensis* | 3 | West mediterranean | 51 | 96 | * |
| **M082** | 03-3559 |  | 2003 | Human | Algeria | *Brucella* | *melitensis* | 3 | West mediterranean | 51 | 92 | j |
| **M083** | 03-4105 |  | 2003 | Human | Maghreb | *Brucella* | *melitensis* | 3 | West mediterranean | 51 | 94 | d |
| **M084** | 02-4188 |  | 2002 | Human | Morocco | *Brucella* | *melitensis* | 3 | West mediterranean | 51 | 92 | * |
| **M085** | 02-8197 |  | 2002 | Human | Maghreb? | *Brucella* | *melitensis* | 3 | West mediterranean | 51 | 94 | 2007AlDahouk#021 ^&^ |
| **M086** | 02-8287 |  | 2002 | Human | Algeria | *Brucella* | *melitensis* | 3 | West mediterranean | 51 | 92 | * |
| **M087** | 96-10539 |  | 1996 | Ovine | Morocco | *Brucella* | *melitensis* | 3 | West mediterranean | 51 | 94 | * |
| **M088** | 92-7524-1 |  | 1992 ? | Human | Algeria | *Brucella* | *melitensis* | 3 | West mediterranean | 51 | 94 | 2006LeFlèche#096 ^&^ |
| **M089** | 89-11818-1 |  | 1989 | Human | Algeria | *Brucella* | *melitensis* | 3 | West mediterranean | 51 | 94 | k |
| **M090** | 89-11818-8 |  | 1989 | Ovine | Algeria | *Brucella* | *melitensis* | 3 | West mediterranean | 51 | 94 | k |
| **M091** | 13-1994-3417 |  | 2013 | Human | Turkey | *Brucella* | *melitensis* | 3 | East mediterranean | 43 | 125 | * |
| **M092** | Ether |  | 1965 | Goat | Reference (Italy) | *Brucella* | *melitensis* | 3 | West mediterranean | 51 | 96 |  |
| **M093** | 16M |  |  | Goat | Reference (USA) | *Brucella* | *melitensis* | 1 | American | 47 | 136 |  |

Table footnotes:

ND: Not determined; *: unique genotype newly described in this study; §: MLVA-11 genotype newly described in this study; $: MLVA-11 genotype newly described in this study; a-l: MLVA-16 genotype harbored by various strains; ^&^: *Brucella*2012 database.
