# Supplementary material for: Human Brucellosis in Maghreb: Existence of a Lineage Related to Socio-Historical Connections with Europe
Source: PLoS One. 2014 Dec 17;9(12):e115319. doi: 10.1371/journal.pone.0115319 (PMC4269447; doi:10.1371/journal.pone.0115319)
Supplement: S2 Table — Data of small ruminant brucellosis reported in Algeria. (DOCX) [file pone.0115319.s003.docx]

Supplementary Table S2 : **Data of small ruminant brucellosis reported in Algeria**

| **Year** | **Occurrence** | **Species** | **Number of** | | | **Number of animals** | | **Total (cap + ovi)** | |
| --- | --- | --- | --- | --- | --- | --- | --- | --- | --- |
|  |  |  | **outbreaks** | **cases** | **deaths** | **destroyed** | **slaughtered** | **outbreaks** | **cases** |
| **2014**  (1st semester) | + | cap | 20 | 87 | 0 | 0 | 87 | 20 | 87 |
|  |  | ovi |  | 0 | 0 | 0 | 0 |  |  |
| **2013** | + | cap | 39 | 110 | 0 | 0 | 110 | 39 | 110 |
|  |  | ovi |  | 0 | 0 | 0 | 0 |  |  |
| **2012** | + | cap | 39 | 140 | 0 | 0 | 140 | 39 | 140 |
|  |  | ovi |  | 0 | 0 | 0 | 0 |  |  |
| **2011** | + | cap | 68 | 355 | 0 | 0 | 355 | 68 | 355 |
|  |  | ovi |  | 0 | 0 | 0 | 0 |  |  |
| **2010** | + | cap | 230 | 1847 | 4 | 0 | 1843 | 230 | 1847 |
|  |  | ovi |  | 0 | 0 | 0 | 0 |  |  |
| **2009** | + | cap | 174 | 979 | 3 | 0 | 976 | 174 | 976 |
|  |  | ovi |  | 0 | 0 | 0 | 0 |  |  |
| **2008** | + | cap | 111 | 481 | 3 | 0 | 478 | 111 | 481 |
|  |  | ovi |  | 0 | 0 | 0 | 0 |  |  |
| **2007** | + | cap | 363 | 1764 | 1 | 0 | 1763 | 363 | 1861 |
|  |  | ovi |  | 97 | 1 | 2 | 94 |  |  |
| **2006** | + | cap | 1253 | 3592 | 2 | 1 | 3589 | 1253 | 3799 |
|  |  | ovi |  | 207 | 0 | 0 | 207 |  |  |
| **2005** | ND | cap | ND | ND | ND |  |  | ND | ND |
|  |  | ovi | ND | ND | ND |  |  |  |  |
| **2004** | + | cap | 709 | 2168 | ND |  | 2168 | 735 | 2290 |
|  |  | ovi | 26 | 122 | ND |  | 122 |  |  |
| **2003** | + | cap | 463 | 2012 | ND |  | 2012 | 473 | 2072 |
|  |  | ovi | 10 | 60 | ND |  | 60 |  |  |
| **2002** | + | cap | 452 | 1552 | 0 |  | 1414 | 473 | 1866 |
|  |  | ovi | 21 | 314 | 0 |  |  |  |  |
| **2001** | + | cap | 510 | 1993 | 0 |  | 1675 | 541 | 2097 |
|  |  | ovi | 31 | 104 | 0 |  |  |  |  |
| **2000** | + | cap | 659 | 2719 | 0 |  | 2065 | 691 | 2898 |
|  |  | ovi | 32 | 179 | 0 |  |  |  |  |
| **1999** | + | cap | 513 | 2295 | 0 |  | 1838 | 540 | 2437 |
|  |  | ovi | 27 | 142 | 0 |  |  |  |  |
| **1998** | + | cap | 528 | 1732 | ND | ND | 1372 | 552 | 1892 |
|  |  | ovi | 24 | 160 | ND | ND | ND |  |  |
| **1997** | + | cap | ND | 3713 | ND | ND | 2708 | 0 | 3898 |
|  |  | ovi | ND | 185 | ND | ND | ND |  |  |
| **1996** | + | cap | ND | 2437 | ND | 1787 | ND | 0 | 2579 |
|  |  | ovi | ND | 142 | ND | ND | ND |  |  |

Table footnotes:

Cases: Animals affected by the disease (sick animals + animals that died from the disease).; Outbreak: Means an occurrence of the disease in question in an agricultural establishment, breeding establishment or premises, including all buildings and all adjoining premises, where animals are present. Where it cannot be defined in this way, the outbreak shall be considered as occurring in the part of the territory in which, taking local conditions into account, it cannot be guaranteed that both susceptible and non-susceptible animals have had no direct contact with affected or suspected cases in that area.; Animals destroyed: Animals killed for disease control purposes whose carcases were destroyed.; Animals slaughtered: Animals killed for disease control purposes with no restrictions on the use of the slaughter products thus obtained.; Deaths: Animals that died from the disease.;

Cap : Goats ; Ovi : Sheep; ND: not determined
